# Diagnostic role of circulating long non-coding RNA LINC00312 in patients with non-small cell lung cancer: a retrospective study

**DOI:** 10.1186/s12885-024-13393-1

**Published:** 2025-01-09

**Authors:** Ruoqian Zhang, Yan Jiang, Jing Gu, Xilin Zhang, Yanping Xie

**Affiliations:** 1 Department of Respiratory Medicine, First Affiliated Hospital of Huzhou University, Huzhou University, Huzhou, Zhejiang 313000 China; 2 Department of Urology Surgery, First Affiliated Hospital of Huzhou University, Huzhou, Zhejiang 313000 China; 3 Central Laboratory, Huzhou Key Laboratory of Translational Medicine, First Affiliated Hospital of Huzhou University, 158 Guangchang Back Road, Huzhou, Zhejiang 313000 P.R. China

**Keywords:** NSCLC, lncRNA, LINC00312, Diagnosis, Exosomes

## Abstract

**Background:**

LINC00312 has shown to play a suppressive role in the development and progression of non-small cell lung cancer (NSCLC). However, the expression pattern and diagnostic role of circulating LINC00312 in NSCLC remain to be confused.

**Methods:**

A total of 319 patients diagnosed with NSCLC and 180 healthy volunteers were enrolled from the First Affiliated Hospital of Huzhou University between January, 2022 and December, 2023. The participates were randomly assigned into the training and validation groups with a ratio of 6:4, while the remaining was named as the exosomal group. Reverse transcription-quantitative PCR (RT-qPCR) was employed to investigate the expression pattern of LINC00312 in NSCLC tissues, serum samples and cell lines. Receiver operating characteristic (ROC) curve analysis was carried out for distinguishing NSCLC from healthy volunteers.

**Results:**

Here, we revealed that LINC00312 was lowly expressed in NSCLC and low LINC00312 expression manifested a poor prognosis. Additionally, compared with the healthy volunteer group, a reduction of circulating LINC00312 in patients with NSCLC was observed in both the training and validation groups. Further correlation analysis indicated that circulating LINC00312 expression was tightly associated with lymph node metastasis, cancer thrombus, spread through air space (STAS) status and pathological type. Moreover, circulating LINC00312 showed a good performance to distinguish NSCLC from healthy volunteers with a higher sensitivity and specificity values. Lastly, exosomal LINC00312 level was also decreased in NSCLC compared with in healthy volunteers.

**Conclusions:**

Taken together, these data unveil that circulating LINC00312 was notably downregulated in NSCLC, offering a novel non-invasive marker for diagnosis of NSCLC.

**Supplementary Information:**

The online version contains supplementary material available at 10.1186/s12885-024-13393-1.

## Introduction

Lung cancer remains the most common deadly malignant tumors [1]. According to the World Cancer Report 2022 [2, 3], the new cases of lung cancer were 2.48 million and the died cases were 1.82 million. Non-small cell lung cancer (NSCLC) is a primary pathological type and mainly consisted of lung adenocarcinoma (LUAD), lung squamous cell carcinoma (LUSC), and large cell carcinoma, accounting for approximately 85% of all lung cancer samples. Due to the lack of early symptoms, patients with NSCLC lose the opportunity of operation at the time of diagnosis [4]. Therefore, it is urgent to excavate effective molecular targets, investigate the molecular mechanisms, and put forward new strategies for the accurate diagnosis of NSCLC.

At present, the low-dose CT combination with serum tumor markers is commonly recommended as an early screening method in clinic. However, the sensitivity and specificity for lung cancer does not exceed 25%, or even less than 10% [5, 6]. On the contrary, long non-coding RNA (lncRNA) is a class of the length longer than 200 nucleotide without protein coding capability [7]. Studies have unveiled that lncRNAs were abnormally expressed in normal and tumor tissues, and their expressions were dramatically associated with tumourigenesis and malignant progression through regulating targeted gene expression [8–12]. In recent years, there has been a rising number studies indicated that lncRNAs were existed and detected in body fluid, and played a prominent role in the diagnosis of cancer [13–15], which acted as novel diagnostic biomarkers for cancer.

LINC00312, located on 3p25.3, is a long intergenic non-coding RNA and uses as a putative tumor suppressor gene [16]. Previous findings have suggested that LINC00312 was abnormally expressed and participated in the development and progression of various solid tumors, including NSCLC [17–22], hepatocellular carcinoma [23], ovarian cancer [24, 25], colorectal cancer [26], breast cancer [27], nasopharyngeal carcinoma [28, 29], head and neck cancer [30], and buccal mucosa cancer [31], suggesting that LINC00312 may be used as a diagnostic marker or anti-tumour target. For instance, the expression of LINC00312 was decreased in NSCLC, and functional experiments indicated that overexpression of LINC00312 was notably inhibited cell proliferation and induced apoptosis of NSCLC in vitro and in vivo [17]. Additionally, Peng et al. [19] implicated that LINC00312 induced metastasis and vasculogenic mimicry via directly binding to the transcription factor YBX1 in lung adenocarcinoma. However, the expression pattern and diagnostic role of circulating LINC00312 in NSCLC remain to be elucidated.

In this study, we observed that LINC00312 is notably reduced both in NSCLC tissues and in cell lines. Additionally, patients with low LINC00312 expression exhibited a poor prognosis. Consistent with the results from NSCLC tissues, circulating LINC00312 level was dramatically decreased in NSCLC compared with in healthy volunteers. Further correlation analysis revealed that circulating LINC00312 level was tightly associated with lymph node metastasis, cancer thrombus, spread through air space (STAS) status and pathological type. Moreover, circulating LINC00312 showed a good performance for distinguishing NSCLC from healthy volunteers compared with CYFRA21-1, which is a common serum tumor marker for NSCLC [32]. Lastly, exosomal LINC00312 was also lowly expressed in NSCLC. Taken together, circulating LINC00312 may be used as a novel and non-invasive diagnostic marker for NSCLC.

## Materials and methods

### Study design and participants

A total of 319 patients diagnosed with NSCLC and 180 healthy volunteers from the First Affiliated Hospital of Huzhou university were enrolled between January, 2022 and December, 2023. The inclusion criteria of participants were contained: ① free of other malignant diseases; ② NSCLC cases were diagnosed by two pathologists based on the 8th Edition of the Union for International Cancer Control guidelines [33]; and ③ voluntarily participated in this study. Participants were excluded from this study when they satisfied one of the following criteria: ① concurrent presence of other malignant diseases; ② insufficient pathological data; and ③ existing serious communication disorders. The above inclusion and exclusion criteria were performed to guarantee the quality and credibility of the study, and minimize potential biases and errors. All samples were collected prior to operation or therapy, and stored at -80˚C for further analysis. Additionally, 16 paired NSCLC and adjacent normal lung tissues were gathered for validating the result of The Cancer Genome Atlas (TCGA) database. Samples were randomly assigned into three groups, including training, validation, and exosomal groups. The training group (147 patients with NSCLC and 94 healthy volunteers) and the validation group (98 patients with NSCLC and 64 healthy volunteers) with a ratio of 6:4, while the remaining cases were exosomal group. Lastly, clinical pathological characteristics and serum tumor marker CYFRA21-1 were selected and analyzed, such as age, gender, smoking history, tumor size, tumor differentiation, TNM stages, lymph node metastasis, cancer thrombus, STAS status, pathological type, and Ki-67 level. The present study was carried out, according to the clinical study protocol provided by the Ethics Committee of the First Affiliated Hospital of Huzhou University (Approval number: 2022KYLL020). The study was also performed in according with the ethical principles of the World Medical Association Declaration of Helsinki (as revised in 2013), and all participants gave written informed consent.

### TCGA database

TCGA database (https://cancergenome.nih.gov/) was used to investigate the expression of all genes in 33 cancer types. Data on the expression level of RNA fragments per kilobase million (FPKM) for a total of 120 cases were downloaded from the TCGA-LUAD (240306_at) dataset. The FPKM values of RNA-Seq data were transformed into TPM (Transcripts per kilobase million). The TPM values were subsequently normalized to log2 for further analysis.

### Kaplan-Meier plotter analysis

The Kaplan-Meier plotter Lung Cancer database (http://kmplot.com/analysis/index.php?p=service&cancer=lung) was employed to investigate the relationship between the expression of genes and survival in lung cancer based on the discovery and validation of survival biomarkers [34]. The entire database contained 2852 tumor cases from 17 independent cohorts (GSE102287, GSE14814, GSE157011, GSE19188, GSE29013, GSE30219, GSE31210, GSE3141, GSE31908, GSE37745, GSE43580, GSE4573, GSE50081, GSE68465, GSE77803, GSE8894, TCGA) [34]. Among them, 2227 patients had overall survival data. Additionally, 1411 patients had overall survival data in the dataset (LINC00312, 240306_at). Based on the median expression of LINC00312, the patients were assigned into higher LINC00312 and lower LINC00312 groups. The overall survival analysis was carried out using a log-rank test [35].

### RNA extraction and reverse transcription-quantitative PCR (RT-qPCR)

TRIzol^®^ reagent was used to extract serum and exosomes total RNA (Invitrogen, Thermo Fisher Scientific, Inc.). Subsequently, the concentration and purity of RNA were assessed using a NanoDrop 2000 (Invitrogen, ThermoFisher Scientific, Inc.). 500ng RNA was reversely transcribed into cDNA using the PrimeScript RT reagent kit (Takara Biotechnology Co., Ltd.), in comparison with the manufacturer’s protocol. The reaction conditions were incubated at 37˚C for 15 min and 85˚C for 5 sec. RT-qPCR was subsequently conducted by the UltraSYBR Mixture (CWBIO, Beijing, China) on an ABI7500 system (Applied Biosystems; Thermo Fisher Scientific, Inc.). The thermocycling conditions used for the RT-qPCR were at 95˚C for 10 min, followed by 40 cycles of 95˚C for 15 sec and 60˚C for 1 min. 18S ribosomal RNA (18sRNA) was used as the internal reference, and the relative expression level was calculated and analyzed using the 2^−ΔΔCt^ method [36]. The primer sequences used in this experiment were as follows: LINC00312 forward, 5’-CTCGCTGGGTTATCAGGCTT-3’; LINC00312 reverse, 5’-TCCTTCAACAGCGTCATCCC-3’; and 18sRNA forward, 5’-GTAACCCGTTGAACCCCATT-3’ and 18sRNA reverse, 5’-CCATCCA ATCGGTAGTAGCG-3’.

### Western blotting

Protein was extracted from exosomes using RIPA buffer containing Protease inhibitor on ice. The protein concentration was quantified by BCA protein detection assay (Beyotime of Biotechnology, Shanghai). An equal amount protein was loaded and separated by SDS-PAGE. Subsequently, the protein was transferred onto the polyvinylidene fluoride (PVDF) membrane, which was blocked with 5% non-fat milk at room temperature for 1 h. The membrane was incubated with primary antibodies overnight at 4˚C and then washed three times with phosphate buffer solution (PBS) containing 0.1% Tween-20, followed by incubated with the corresponding secondary antibody at room temperature for 1 h. The images were obtained and visualized using enhanced ECL chemiluminescence assay kit (Beyotime of Biotechnology) via the Tanon-5200. The primary antibodies utilized in this experiment were as follows: anti-Alix (dilution 1:1000; Cat No. 12422-1-AP, Proteintech); anti-TSG101 (dilution 1:1000; Cat No. 28283-1-AP, Proteintech); anti-CD9 (dilution 1:1000; Cat No. 20597-1-AP, Proteintech); and anti-β-tubulin (dilution 1:5000; Cat No. 10094-1-AP, Proteintech).

### Exosomes isolation

Exosomes were isolated from serum samples using Hieff^®^ Quick exosome isolation kit (for serum/plasma) (YEASEN Biotechnology Co., Ltd.), in accordance with the manufacturer’s protocol and previous report [37]. Briefly, 1 ml serum samples were sequentially centrifuged at 3,000 g for 10 min and 10,000 g for 20 min. The supernatant was diluted with PBS at a ratio of 1:4. The exosome isolation kit was subsequently added, mixed, and stewing overnight at 4˚C. Afterward, exosomes were obtained by centrifugation at 10,000 g for 1 h and stored at -80˚C for further use.

### Electron microscopy

Exosomes were identified by electron microscopy, in accordance with the previous study [38]. Briefly, 15 µl exosomes were loaded onto the copper mesh and stained with 2% uranyl acetate solution for 1 min at room temperature, followed by washing and drying. The images were carried out under a Tecnai G2 spititi electron microscopy.

### Nanoparticle tracking analysis (NTA)

Nanoparticle tracking analysis was employed to detect the size distribution and concentration of exosomes, in accordance with the manufacturer’s instruction. The exosomes were diluted at 1:100 in PBS solution, and then the particles of exosomes were observed under a NanoSight NS3000 Instrument (Malvern Instrument, Ltd.).

### Statistical analysis

A calculation based on the formula for epidemiological cross-sectional studies was conducted to estimate the sample size for this study. The formula is as follows: n = $\:\frac{{z}_{1-\alpha\:/2}^{2}\:*\:P(1-P)}{{d}^{2}}$ * (1 + 20%). Among them, n represents the sample number, z is the statistic, 1-α/2 is the two-sided test, *P* is the diagnostic rate of serum tumor markers, and d is the precision. Previous studies have revealed that the occurrence of serum tumor markers for diagnosis of NSCLC is about 25% [39]. Postulating a value of confidence internal is 95% and a value of d is 5% as well as a two-sided test and a 20% lost to follow-up rate, the number of this study was calculated to be at least 286 samples. Lastly, 319 NSCLC patients was enrolled in the present study.

All data were presented as the mean ± standard deviation (SD). Data analysis was performed by GraphPad Prism 6.0 software (GraphPad Software, San Diego, CA, USA). The statistical analyses of two groups and multiple groups were performed using Student’s *t*-test and one-way analysis of variance (ANOVA), respectively. Spearman’s correlation analysis was employed to evaluate the relationship between LINC00312 expression and clinicopathological characteristics of patients with NSCLC. The overall survival was carried out using Kaplan-Meier analysis with a log-rank test. Receiver operating characteristic (ROC) curve analysis was established to assess the sensitivity and specificity of LINC00312 and CYFRA21-1 for diagnosis of NSCLC. A *P*-value of < 0.05 was considered to indicate a statistically significant.

## Result

### Clinical significance of LINC00312 in NSCLC

**Fig. 1:**
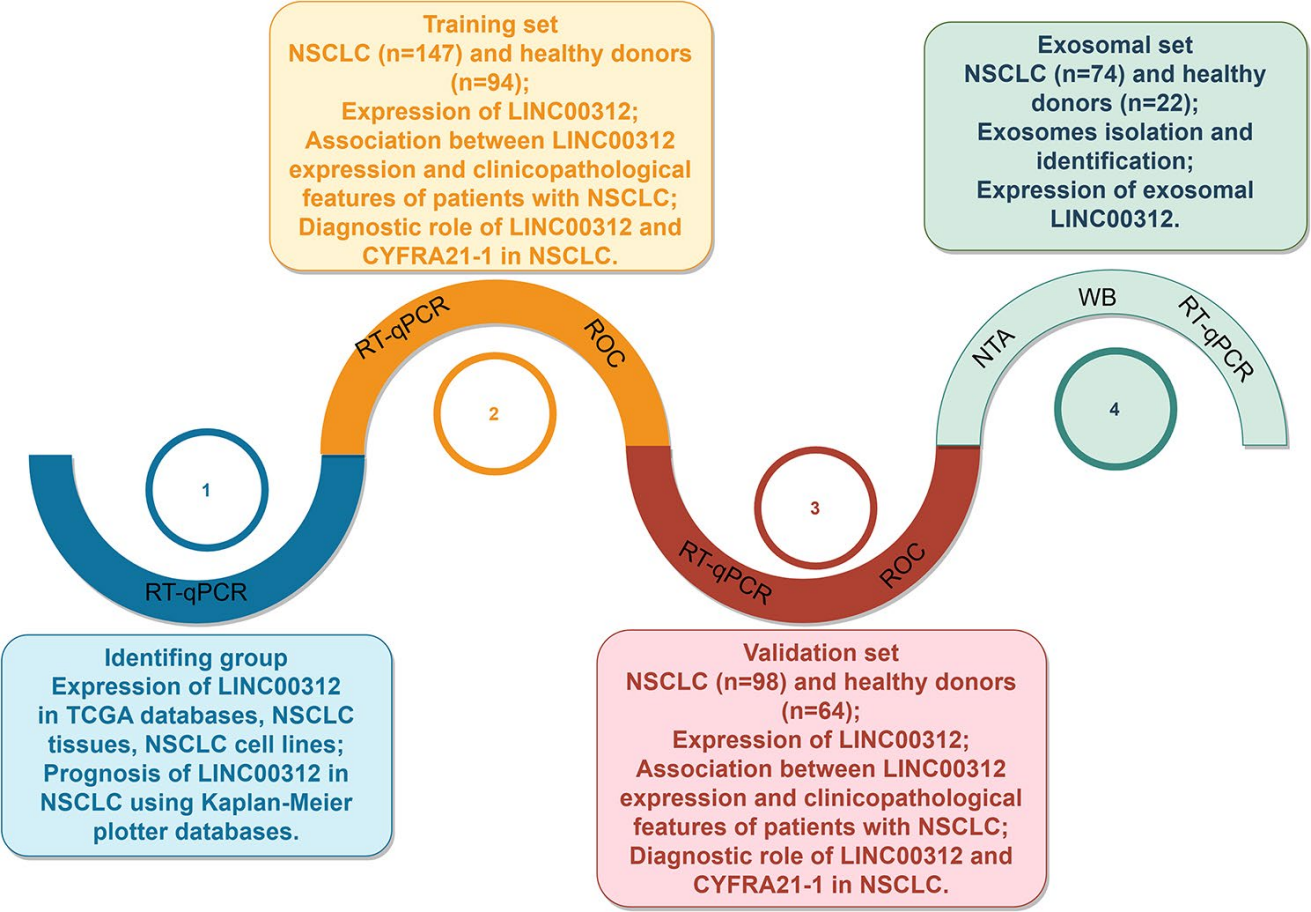
A flow diagram of the present study. The present study was mainly consisted of four parts, including identification, training, validation and exosomal groups. All serum samples were randomly assigned to three groups, including training, validation and exosomal groups

**Fig. 2:**
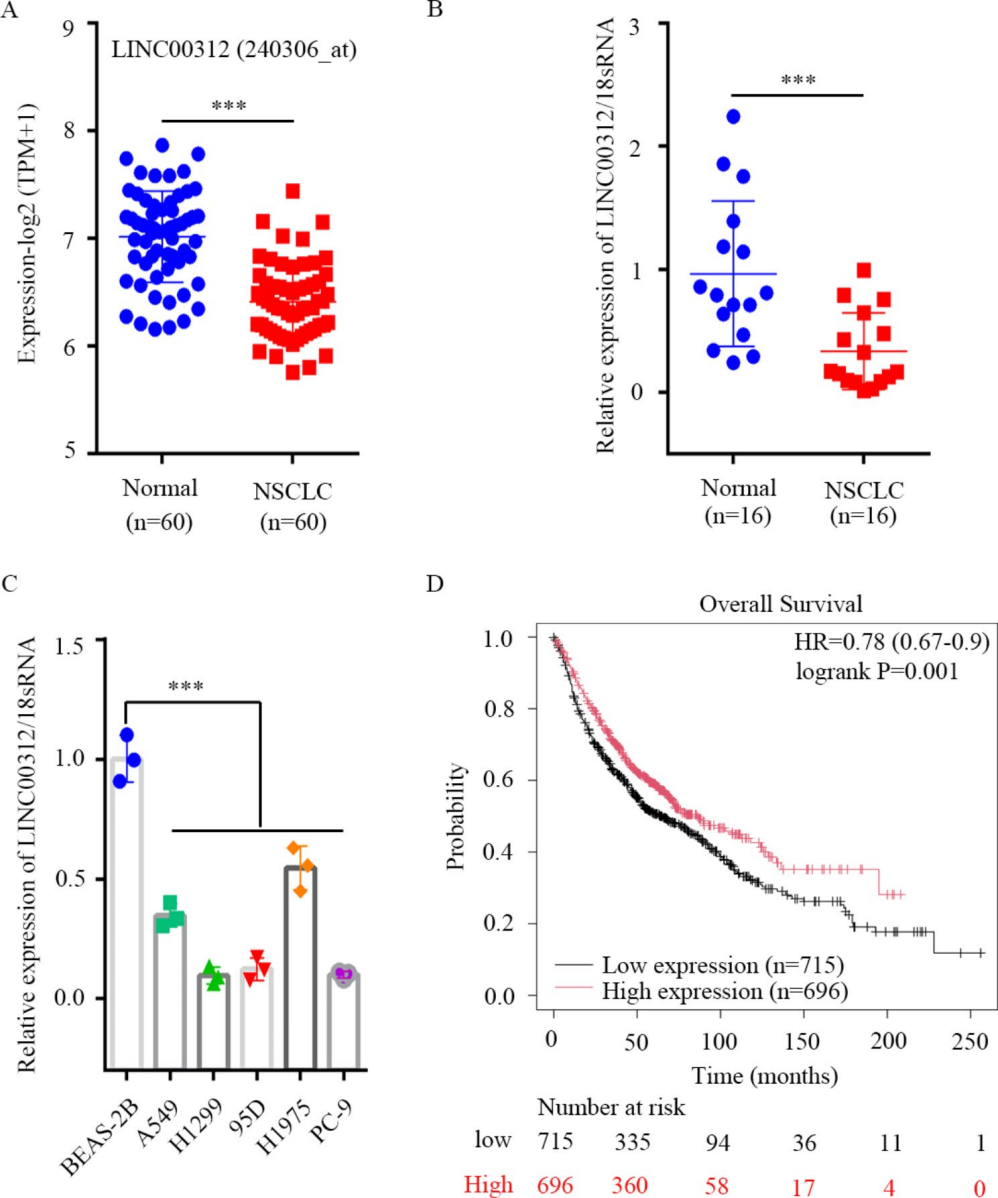
Clinical significance of LINC00312 expression in NSCLC. **A**: TCGA database (240306_at) was downloaded and analyzed the expression of LINC00312 in tumor and matched lung normal tissues, *n* = 60. **B**: RT-qPCR was employed to determine the expression of LINC00312 in NSCLC tumor and adjacent normal tissues, *n* = 16. **C**: LINC00312 level was measured in five NSCLC cell lines and normal lung epithelial cell (BEAS-2B) using RT-qPCR. **D**: Overall survival probability of two groups based on the median expression level of LINC00312 using the Kaplan-Meier plotter database. *** represents significant difference at *P* < 0.001

As indicated in Fig. 1, a flow diagram of the present study. This study was probably composed of four parts, including identification, training, validation, and exosomal groups. The purpose of this study was designed to unveil the expression pattern and diagnostic role of circulating LINC00312 in metastatic NSCLC, providing a novel non-invasive marker for NSCLC. To investigate the expression pattern of LINC00312 in NSCLC, the TCGA database was downloaded and analyzed. It was observed that LINC00312 level was notably decreased in NSCLC tissues compared with in the matched lung normal tissues (Fig. 2A). To confirmed the result of the TCGA database, the mRNA levels of LINC00312 were detected in 16 paired NSCLC tissues and adjacent normal lung tissues using RT-qPCR. It was also observed that LINC00312 was lowly expressed in NSCLC (Fig. 2B). Additionally, the mRNA expression levels of LINC00312 in several NSCLC cell lines were both notably reduced than that in normal epithelial cell line BEAS-2B (Fig. 2C). Furthermore, patients with lower LINC00312 expression had a shorter overall survival (*n* = 1411, HR = 0.78, *P* = 0.001) than that in patients with higher LINC00312 expression (Fig. 2D).

### Expression pattern of circulating LINC00312 in NSCLC

**Fig. 3:**
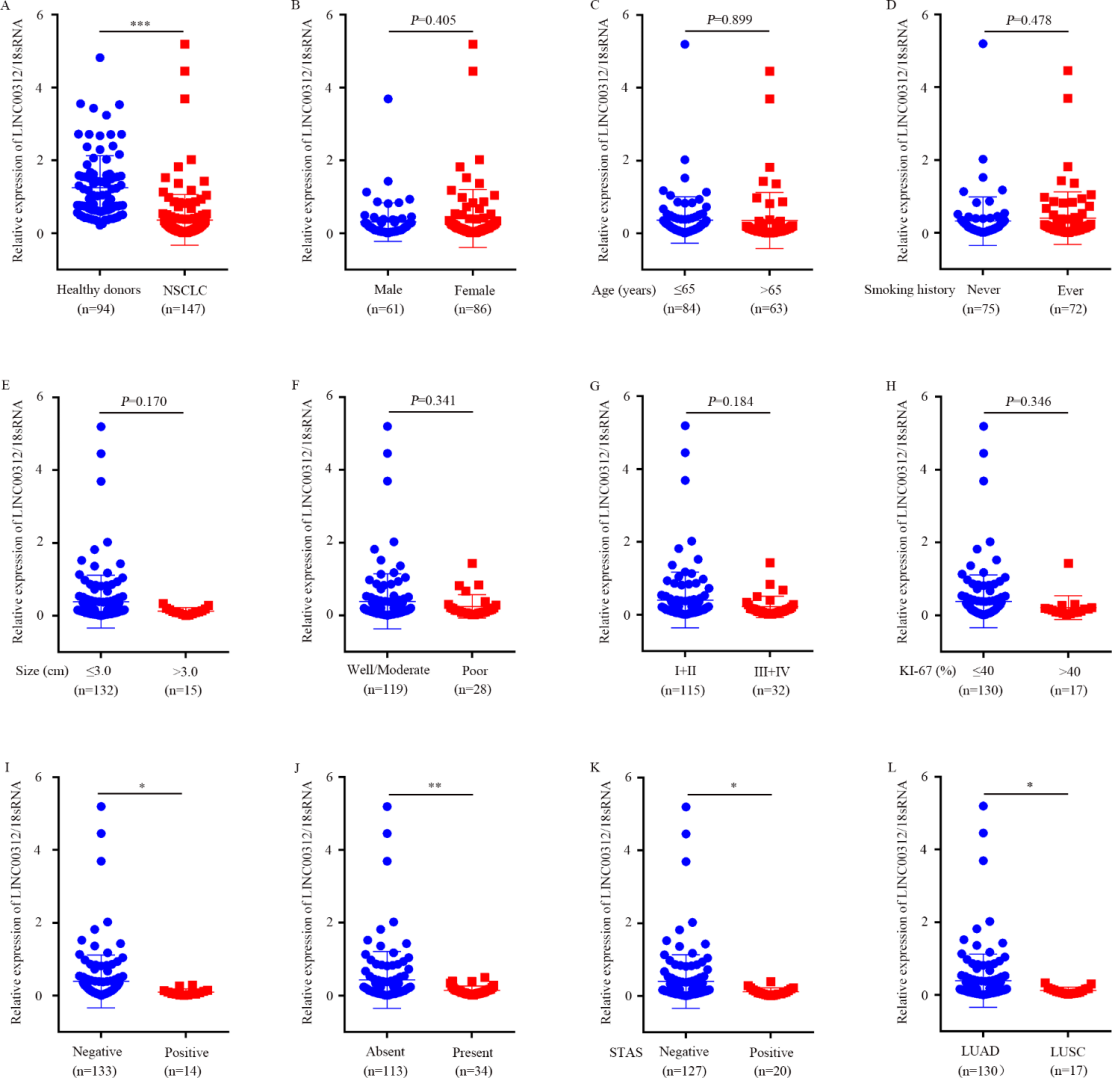
Expression of circulating LINC00312 in the training group. **A**: A total of 147 patients with NSCLC and 94 healthy control serum samples were used as the training set. The circulating LINC00312 expression level was detected using RT-qPCR. Associations between circulating LINC00312 level and clinicopathological characteristics of patients with NSCLC were performed, **B**: Gender; **C**: Age; **D**: Smoking history; **E**: Tumor size; **F**: Tumor differentiation; **G**: TNM stages; **H**: Ki-67 expression; **I**: Lymph node metastasis; **J**: Cancer thrombus; **K**: STAS; **L**: Pathological type. *, **, and *** represent significant differences at *P* < 0.05, *P* < 0.01, and *P* < 0.001, respectively. LUAD, lung adenocarcinoma; LUSC, lung squamous cell carcinoma

**Fig. 4:**
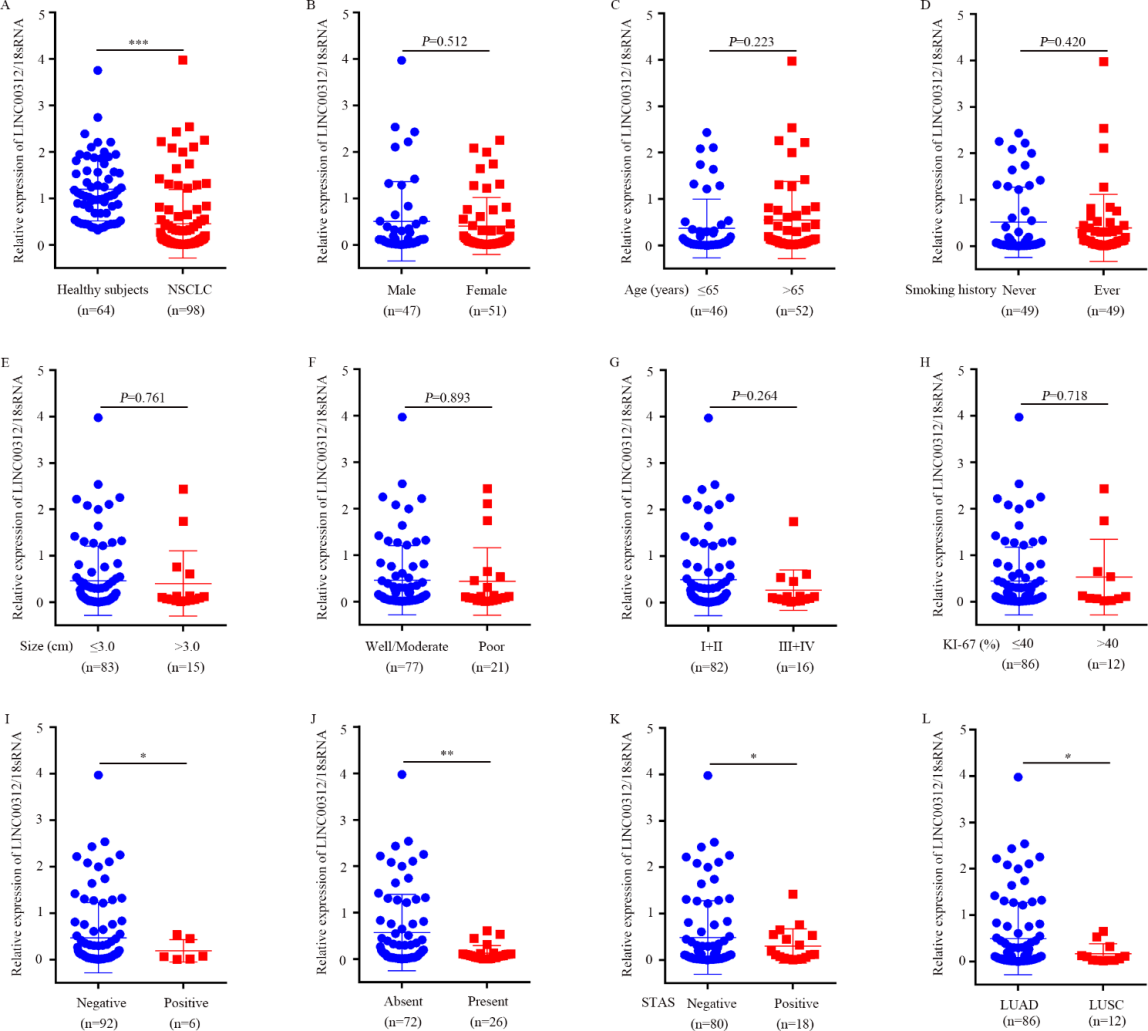
Expression of circulating LINC00312 in the validation group. **A**: A total of 98 patients with NSCLC and 64 healthy control serum samples were recognized as the validation set. The circulating LINC00312 expression level was detected using RT-qPCR. Associations between circulating LINC00312 level and clinicopathological characteristics of patients with NSCLC were conducted, **B**: Gender; **C**: Age; **D**: Smoking history; **E**: Tumor size; **F**: Tumor differentiation; **G**: TNM stages; **H**: Ki-67 expression; **I**: Lymph node metastasis; **J**: Cancer thrombus; **K**: STAS; **L**: Pathological type. *, **, and *** represent significant differences at *P* < 0.05, *P* < 0.01, and *P* < 0.001, respectively. LUAD, lung adenocarcinoma; LUSC, lung squamous cell carcinoma

To explore the expression pattern of circulating LINC00312 in NSCLC, 319 patients with NSCLC and 180 age-matched healthy volunteer serum samples were randomly separated into three groups, including training (147 patients and 94 healthy donors), validation (98 patients and 64 healthy donors) and exosomal (74 patients and 22 healthy donors) groups. Firstly, we quantified the LINC00312 expression levels in the training group using RT-qPCR. Downregulation of LINC00312 was observed in patients with NSCLC than in healthy donors (Fig. 3A). Additionally, the associations between circulating LINC00312 expression and clinicopathological characteristics of patients with NSCLC were performed. The findings revealed that patients with positive lymph node metastasis, present cancer thrombus, positive STAS, and LUSC showed a lower circulating LINC00312 expression than in patients with negative lymph node metastasis, absent cancer thrombus, negative STAS, and LUAD (Fig. 3I-L; Table 1). However, there were not significant associations with age, gender, smoking history, tumor size, tumor differentiation, TNM stages, and Ki-67 expression (Fig. 3B-H; Table 1). Lastly, a validation cohort was employed to validate these results, and proved that circulating LINC00312 expression was exactly downregulated in NSCLC (Fig. 4A), in consistent with the results of the training group. Further relationship analysis indicated that circulating LINC00312 expression was strongly associated with lymph node metastasis, cancer thrombus, STAS status, and pathological type, but not with age, gender, smoking history, tumor size, tumor differentiation, TNM stages, and Ki-67 expression (Fig. 4B-L; Table 2).

**Table 1:** Association between LINC00312 expression and clinicopathological characteristics of patients with non-small cell lung cancer

Features	*N* = 147	LINC00312 expression (Mean ± SD)	t	*P*
Gender			0.836	0.405
Male	61	0.30 ± 0.07		
Female	86	0.40 ± 0.09		
Age (years)			0.128	0.899
> 65	63	0.35 ± 0.10		
≤ 65	84	0.37 ± 0.07		
Smoking history			0.712	0.478
Never	75	0.32 ± 0.08		
Ever	72	0.40 ± 0.09		
Tumor size (cm)			1.381	0.170
> 3.0	14	0.16 ± 0.02		
≤ 3.0	133	0.39 ± 0.06		
Tumor differentiation			0.955	0.341
Well/Moderate	119	0.39 ± 0.07		
Poor	28	0.25 ± 0.06		
TNM stages			1.336	0.184
I + II	115	0.40 ± 0.07		
III + IV	32	0.22 ± 0.05		
Lymph node metastasis			2.103	0.037
Positive	14	0.10 ± 0.02		
Negative	133	0.39 ± 0.06		
Cancer thrombus			2.581	0.009
Present	34	0.14 ± 0.02		
Absent	113	0.43 ± 0.07		
Ki−67 expression (%)			0.945	0.346
> 40	18	0.21 ± 0.08		
≤ 40	129	0.38 ± 0.06		
STAS			1.874	0.046
Positive	20	0.12 ± 0.02		
Negative	127	0.40 ± 0.07		
Pathological type			1.808	0.048
LUAD	130	0.12 ± 0.02		
LUSC	17	0.39 ± 0.06		

**Table 2:** Association between LINC00312 expression and clinicopathological characteristics of patients with non-small cell lung cancer

Features	*N* = 98	LINC00312 expression (Mean ± SD)	t	*P*
Gender			0.658	0.512
Male	47	0.50 ± 0.12		
Female	51	0.41 ± 0.09		
Age (years)			1.228	0.223
> 65	46	0.55 ± 0.12		
≤ 65	52	0.37 ± 0.09		
Smoking history			0.810	0.420
Never	49	0.51 ± 0.11		
Ever	49	0.39 ± 0.10		
Tumor size (cm)			0.305	0.761
> 3.0	15	0.40 ± 0.18		
≤ 3.0	83	0.46 ± 0.08		
Tumor differentiation			0.134	0.893
Well/Moderate	77	0.46 ± 0.08		
Poor	21	0.43 ± 0.16		
TNM stages			1.124	0.264
I + II	82	0.49 ± 0.09		
III + IV	16	0.26 ± 0.11		
Lymph node metastasis			1.907	0.036
Present	6	0.19 ± 0.09		
Absent	92	0.47 ± 0.07		
Cancer thrombus			2.724	0.008
Present	26	0.13 ± 0.03		
Absent	72	0.57 ± 0.10		
Ki−67 expression (%)			0.363	0.718
> 40	12	0.53 ± 0.08		
≤ 40	86	0.44 ± 0.08		
STAS			1.811	0.042
Positive	18	0.15 ± 0.05		
Negative	80	0.52 ± 0.09		
Pathological type			1.829	0.04
LUAD	86	0.49 ± 0.08		
LUSC	12	0.17 ± 0.06		

### Diagnostic value of circulating LINC00312 in NSCLC

**Fig. 5:**
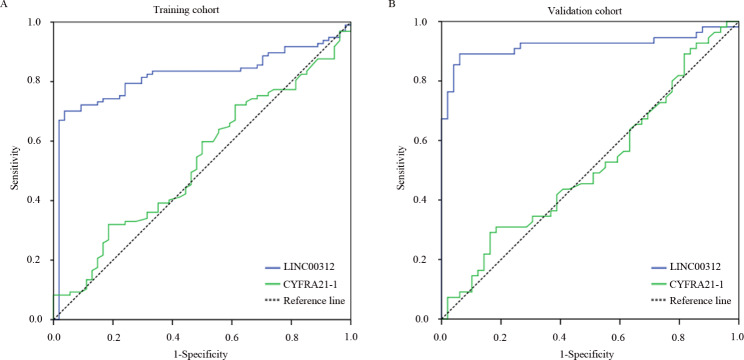
Diagnostic role of circulating LINC00312 in NSCLC. ROC curve analysis was performed to detect the sensitivity and specificity values of circulating LINC00312 and CYFRA21-1 in NSCLC, **A**: Training group; **B**: Validation group

To further investigate the diagnostic performance of circulating LINC00312 in NSCLC, ROC curve analysis was conducted. We observed circulating LINC00312 showed a good performance for distinguishing NSCLC from healthy donors, with an area under curve (AUC) of 0.819 (95% confidence interval [CI]: 0.748–0.890) for the training group and 0.920 (95% CI: 0.859–0.982) for validation group (Fig. 5A and B; Table 3). Additionally, the sensitivity and specificity of circulating LINC00312 were 0.701 and 0.963 for the training group, and 0.855 and 0.959 for the validation group, respectively (Fig. 5A and B). In contrast, the AUC of CYFRA21-1 was 0.532 (95% CI: 0.437–0.627) and 0.516 (95% CI: 0.404–0.628), respectively (Fig. 5A and B; Table 3). The sensitivity value reached 0.361 and 0.436, and specificity value was 0.685 and 0.592, respectively (Fig. 5A and B). These findings revealed that circulating LINC00312 could used as a non-invasive marker for the diagnosis of NSCLC.

**Table 3:** Diagnostic performance of circulating LINC00312 and CYFRA21-1 in patients with non-small cell lung cancer

Training group			
Variables	Area	95% confidence interval (CI)	*P*
LINC00312	0.819	0.748–0.890	< 0.001
CYFRA21−1	0.532	0.437–0.627	0.514
Validation group			
Variables	Area	95% confidence interval (CI)	*P*
LINC00312	0.920	0.859–0.982	< 0.001
CYFRA21−1	0.516	0.404–0.628	0.777

### Expression of exosomal LINC00312 in patients with NSCLC

**Fig. 6:**
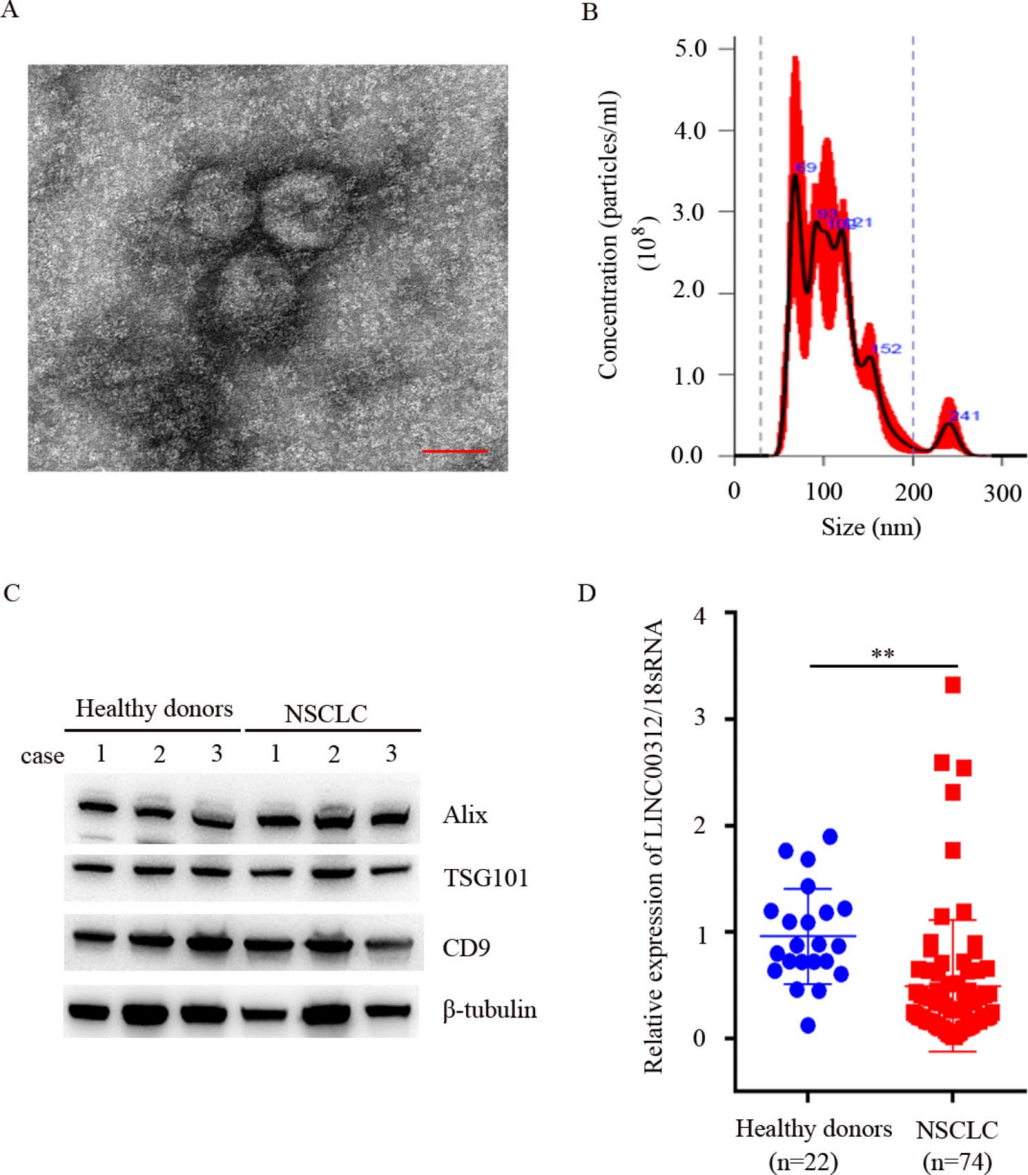
Expression of exosomal LINC00312 in the exosomal group. Exosomes were isolated from patients with NSCLC using the Hieff™ Quick exosome isolation kit (for Serum/Plasma), in comparison with the manufacturer protocol. **A**: Identification of exosomes by electron microscopy; **B**: The size and distribution of exosomes were detected using NTA; **C**: The protein expression of exosomal markers, including CD9, TSG101, and Alix was analyzed using western blotting; **D**: Exosomal LINC00312 expression levels were measured using RT-qPCR. *** represents significant difference at *P* < 0.001. NTA, Nanoparticle tracking analysis

Exosomes were abundantly existed in all bodies fluids. Exosomes were separated and identified using electron microscopy, NTA and western blotting. The data indicated that the size of exosomes was distributed from 65 nm to 152 nm (Fig. 6A and B). The exosomal protein markers were notably detected (Fig. 6C). RT-qPCR was conducted to detect the expression of exosomal LINC00312 in NSCLC and healthy donors. Reduced expression of exosomal LINC00312 was observed in patients with NSCLC than in healthy donors (Fig. 6D). These results implied that circulating LINC00312 may be primary incorporated into exosomes and offered a novel diagnostic marker for NSCLC.

## Discussion

With the widespread application of low-dose chest CT in clinical practice, patients with NSCLC were diagnosed in its early stages [40]. But the majority of patients were belonged to advanced stages at the time of diagnosis, attributing to poor sensitivity and specificity of low-dose chest CT [41]. Considering the important roles in the therapeutic scheme (surgical operation or not) and prognosis predicting, it is urgent to screen novel indicators to achieve early diagnosis and predicting prognosis of NSCLC.

In recent years, there has been a rising number studies have confirmed that abnormal expressions of lncRNAs were notably associated with tumor cell proliferation, migration, invasion, epithelial-mesenchymal transition, apoptosis, DNA damage, and drug resistance [42–46], which used as indicators for diagnostic marker and prognosis predicting of cancer. In the present study, we observed that LINC00312 was notably reduced both in NSCLC tissues and in cell lines, which was consistent with the previous reports [17, 22]. In view of the limitations of direct detection of lncRNAs in NSCLC tissues in clinic, researchers tried to detect the expression of circulating lncRNAs for the diagnosis and prognosis forecasting of NSCLC. It was observed that the changes of lncRNAs in peripheral blood were consistent with in tumor tissues, emphasizing they are promising markers for early diagnosis of NSCLC [15]. For instance, lncRNA HOTAIR was significantly promoted in NSCLC and exhibited a good performance for identifying NSCLC [47]. Elevating circulating GAS5 could discriminate NSCLC from healthy donors with a AUC of 0.81 [13]. Our results unveiled that circulating LINC00312 in NSCLC was dramatically decreased both in the training and validation groups. Further correlation analysis assumed that circulating LINC00312 level was highly associated with positive lymph node metastasis, present cancer thrombus, positive STAS and pathological type, manifesting LINC00312 might be a metastasis indicator. Previous study also revealed that LINC00312 induces lung adenocarcinoma metastasis and vasculogenic mimicry through directly binding to YBX1 [19]. Additionally, circulating LINC00312 emerged a good performance for identifying NSCLC from healthy donors, with an AUC of 0.819 for the training group and 0.920 for the validation group. Sulewska et al. [21] demonstrated that a signature of 14 lncRNAs (HAGLR, ADAMTS9-AS2, LINC00261, MCM3AP-AS1, TP53TG1, C14orf132, LINC00968, LINC00312, TP73-AS1, LOC344887, LINC00673, SOX2-OT, AFAP1-AS1, LOC730101) was a useful diagnostic marker with an AUC of 0.98, suggesting the diagnostic accuracy of lncRNAs panel proves to be better than a standalone marker. These results implied that circulating LINC00312 is a non-invasive diagnostic marker for NSCLC, especially for metastasis NSCLC.

Exosomes, a type of extracellular vesicles, can be stably detected in a variety of biological fluids, such as blood, urine and saliva [48]. Its size was ranged from 30 to 200 nm. Mounting evidence exhibited that exosomes encapsulated proteins, mRNAs, microRNAs, lncRNAs, lipids, etc., which were recognized as novel indicators for diagnostic marker and prognosis predicting of NSCLC [14, 48, 49]. Our previous study reported that exosomal DLX6-AS1 was highly expressed in NSCLC, which was used as a potential marker for early diagnosis of NSCLC [50]. The lncRNA AL139294.1 arises as a notable player in NSCLC, as evidenced by its dramatic upregulation in exosomes of patients with NSCLC [51]. Higher exosomal LUCAT1 expression was consistently associated with shorter overall survival, larger tumor size, positive lymph node metastasis, and advanced stages [52]. In this study, we observed that exosomal LINC00312 was exactly decreased in NSCLC compared with in healthy donors, which was consistent with the findings of serum samples. Concurrently, the present study unveiled that circulating LINC00312 was lowly expressed in NSCLC and exhibited a good performance for identifying NSCLC, offering a novel diagnostic marker for NSCLC. However, this study also exists some limitations. For instance, ① validation group samples were enrolled from our hospital and existed a potential bias in the sample collection. Multi-center samples will be selected for confirming these findings; ② more clinical information should be analyzed, including EGFR and KRAS mutation status, EML4-ALK translocation, PD-L1 expression, which will contribute to emphasize the clinical relevance of circulating LINC00312 in NSCLC; ③ the diagnostic value of LINC00312 combination with existing serum biomarkers will be investigated. More samples will obtain to determine the critical value of LINC00312, offering a diagnostic indicator for early diagnosis of NSCLC. The function and mechanism of exosomal LINC00312 in NSCLC progression remain to be confused. Our future investigations will focus on elucidating the roles and molecular mechanisms of exosomal LINC00312 in mediating the tumorigenesis and progression of NSCLC.

## Electronic supplementary material

Below is the link to the electronic supplementary material.

Supplementary Material 1

## Data Availability

The data are available on reasonable request to the corresponding authors.
