# Supplementary material for: Diagnostic role of circulating long non-coding RNA LINC00312 in patients with non-small cell lung cancer: a retrospective study
Source: BMC Cancer. 2025 Jan 9;25:47. doi: 10.1186/s12885-024-13393-1 (PMC11721336; doi:10.1186/s12885-024-13393-1)

Supplementary Figure legend

Exosomes were isolated from three healthy donor and patients with NSCLC serum samples. The exosomal protein markers were detected using western blotting. A: Alix; B: TSG101; C: CD9; D: β-tubulin. C1, C2 and C3 represent healthy donors; P1, P2 and P3 represent patients with NSCLC.


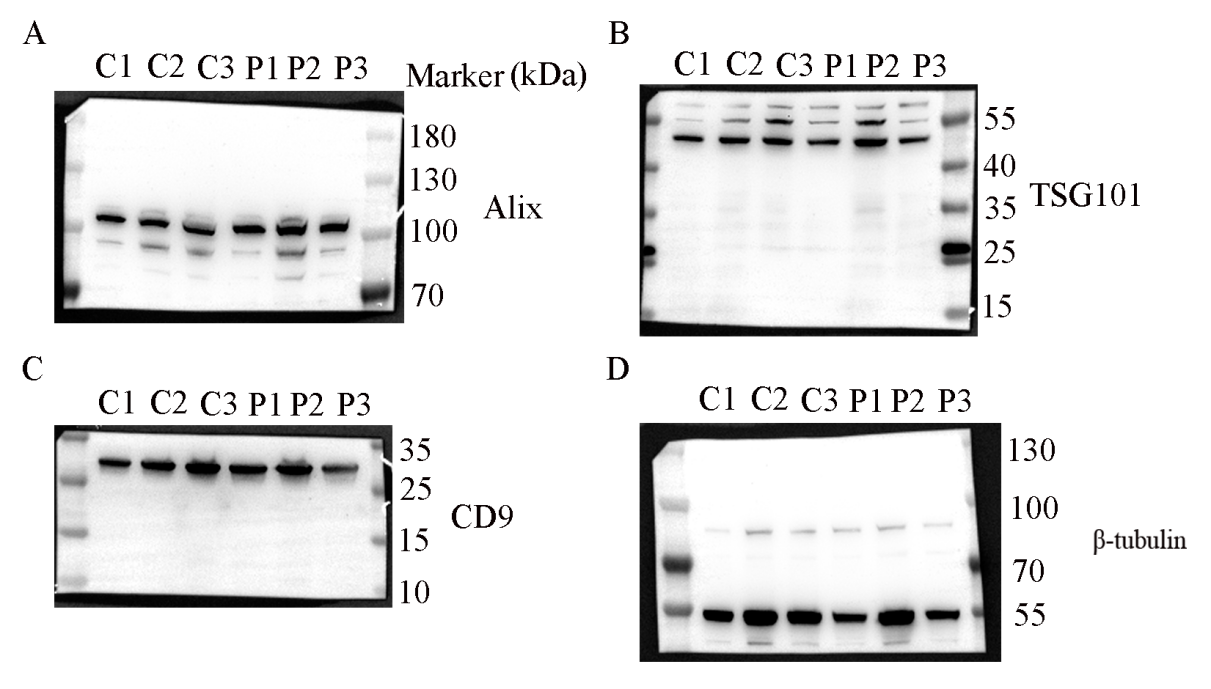

Supplement: Supplementary file 1 — Supplementary Material 1 [file 12885_2024_13393_MOESM1_ESM.docx]
